# Supplementary material for: miR-146a-5p Promotes Angiogenesis and Confers Trastuzumab Resistance in HER2+ Breast Cancer
Source: Cancers (Basel). 2023 Apr 4;15(7):2138. doi: 10.3390/cancers15072138 (PMC10093389; doi:10.3390/cancers15072138)
Supplement: Supplementary file 1 [file cancers-15-02138-s001.zip › cancers-2153920-supplementary.pptx]

## Slide 1
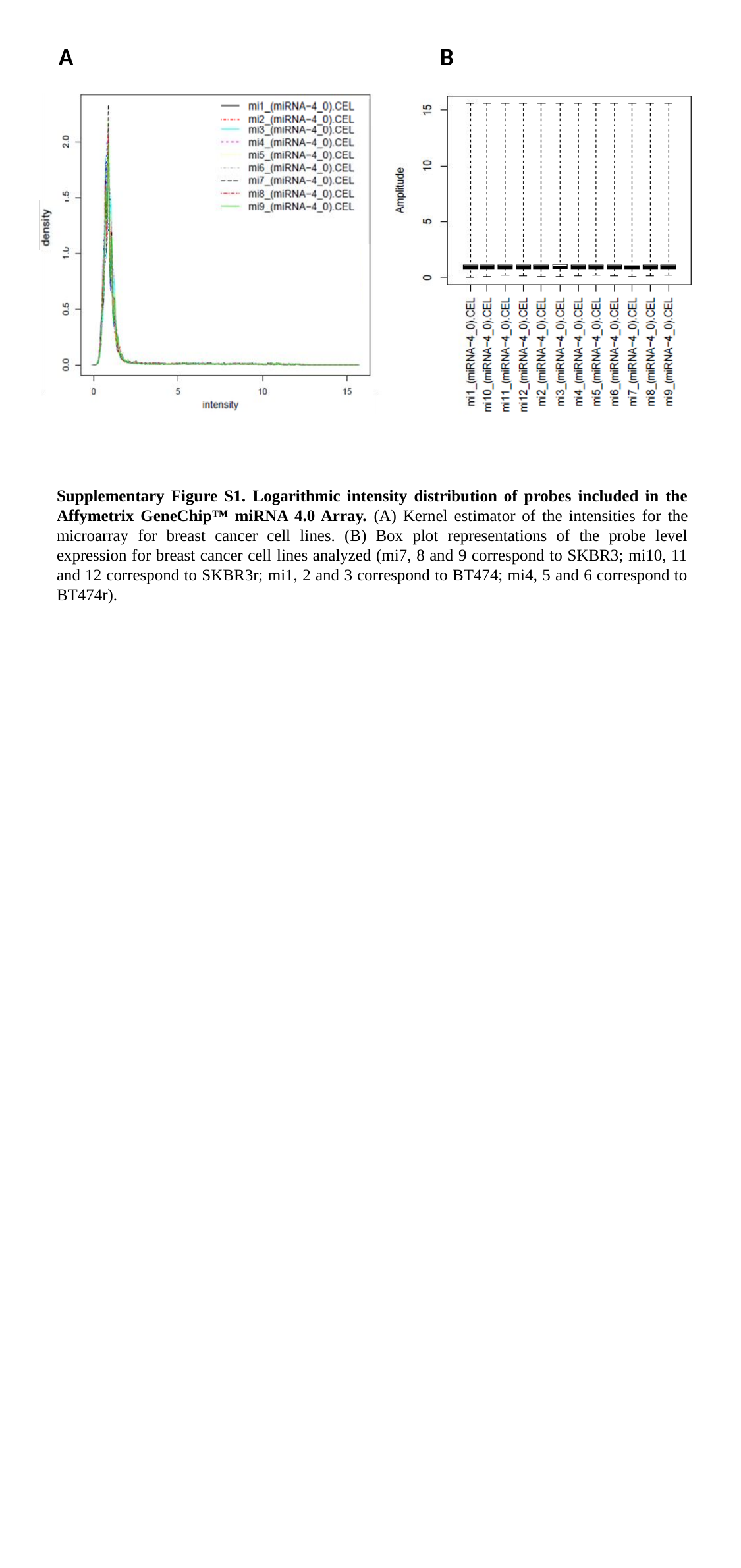

A
B
Supplementary Figure S1. Logarithmic intensity distribution of probes included in the Affymetrix GeneChip™ miRNA 4.0 Array. (A) Kernel estimator of the intensities for the microarray for breast cancer cell lines. (B) Box plot representations of the probe level expression for breast cancer cell lines analyzed (mi7, 8 and 9 correspond to SKBR3; mi10, 11 and 12 correspond to SKBR3r; mi1, 2 and 3 correspond to BT474; mi4, 5 and 6 correspond to BT474r).

## Slide 2
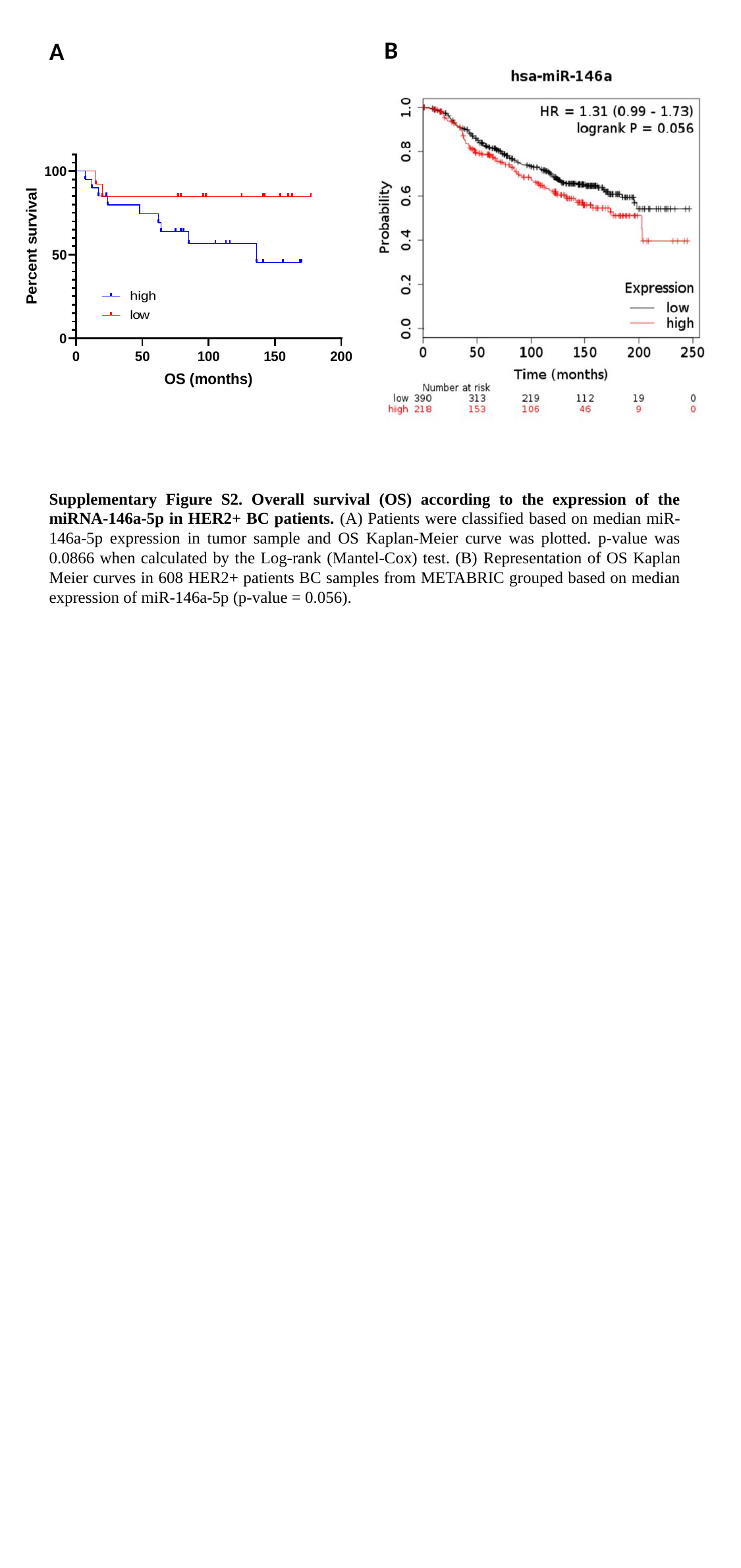

B
A
Supplementary Figure S2. Overall survival (OS) according to the expression of the miRNA-146a-5p in HER2+ BC patients. (A) Patients were classified based on median miR-146a-5p expression in tumor sample and OS Kaplan-Meier curve was plotted. p-value was 0.0866 when calculated by the Log-rank (Mantel-Cox) test. (B) Representation of OS Kaplan Meier curves in 608 HER2+ patients BC samples from METABRIC grouped based on median expression of miR-146a-5p (p-value = 0.056).

## Slide 3
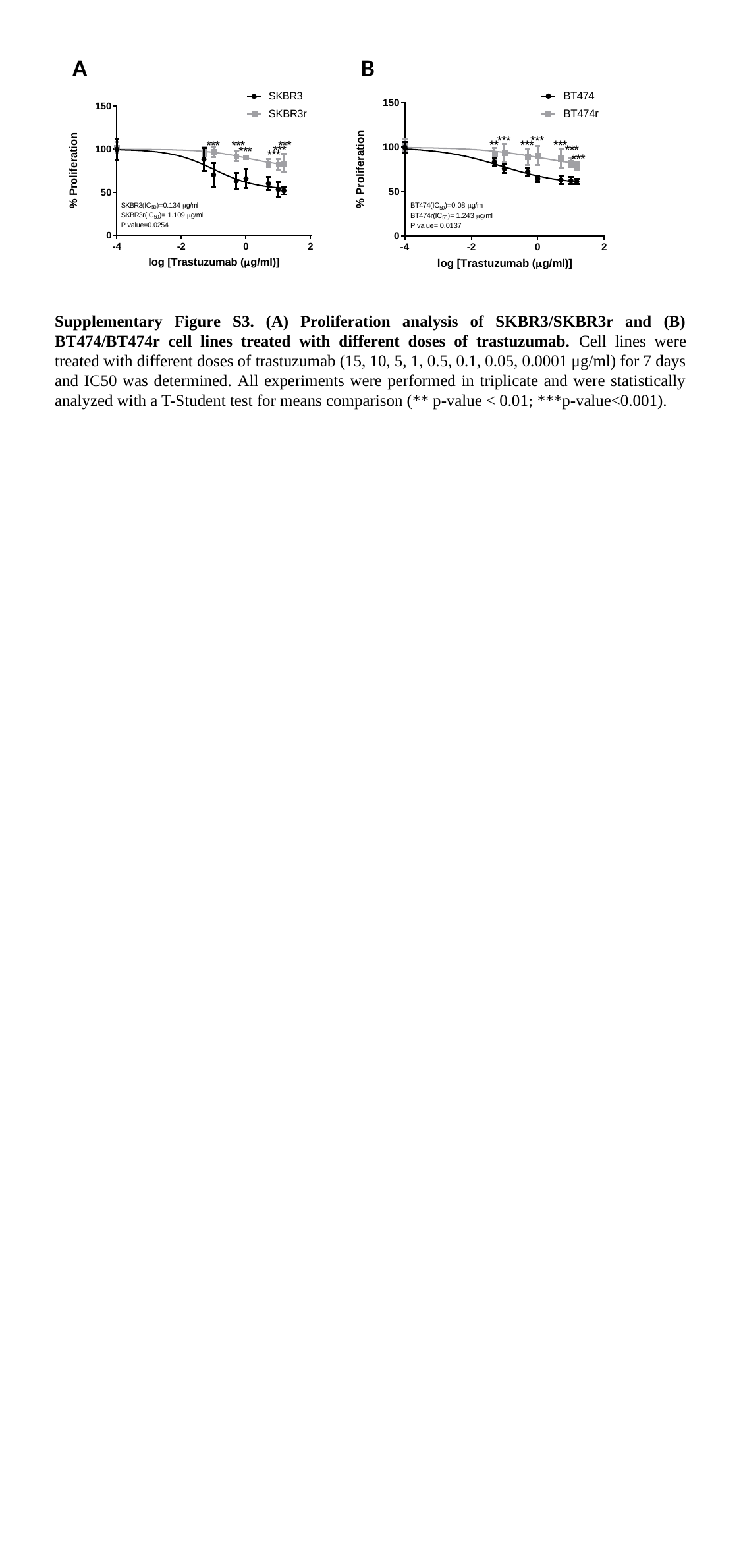

A
B
Supplementary Figure S3. (A) Proliferation analysis of SKBR3/SKBR3r and (B) BT474/BT474r cell lines treated with different doses of trastuzumab. Cell lines were treated with different doses of trastuzumab (15, 10, 5, 1, 0.5, 0.1, 0.05, 0.0001 μg/ml) for 7 days and IC50 was determined. All experiments were performed in triplicate and were statistically analyzed with a T-Student test for means comparison (** p-value < 0.01; ***p-value<0.001).

## Slide 4
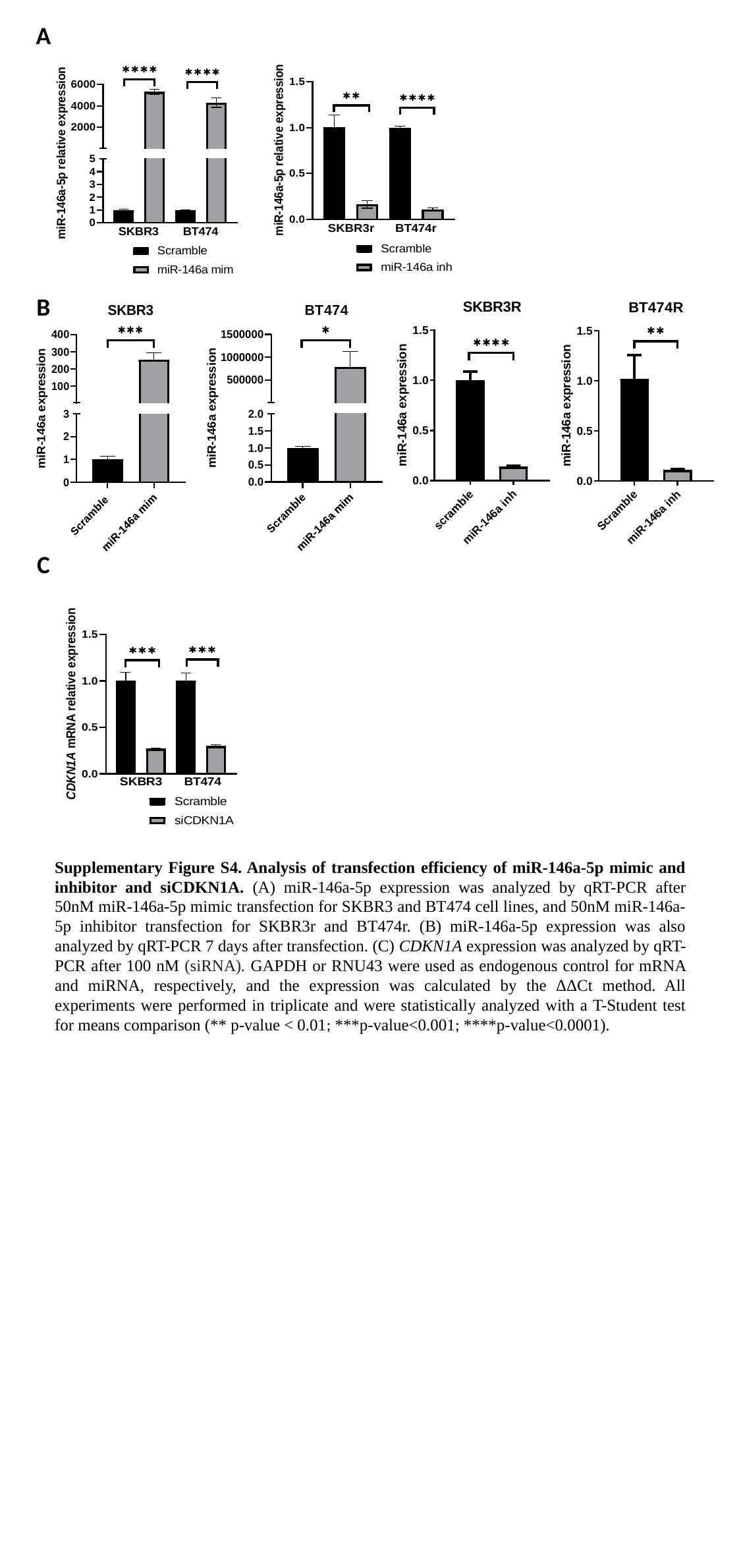

A
B
C
Supplementary Figure S4. Analysis of transfection efficiency of miR-146a-5p mimic and inhibitor and siCDKN1A. (A) miR-146a-5p expression was analyzed by qRT-PCR after 50nM miR-146a-5p mimic transfection for SKBR3 and BT474 cell lines, and 50nM miR-146a-5p inhibitor transfection for SKBR3r and BT474r. (B) miR-146a-5p expression was also analyzed by qRT-PCR 7 days after transfection. (C) CDKN1A expression was analyzed by qRT-PCR after 100 nM (siRNA). GAPDH or RNU43 were used as endogenous control for mRNA and miRNA, respectively, and the expression was calculated by the ΔΔCt method. All experiments were performed in triplicate and were statistically analyzed with a T-Student test for means comparison (** p-value < 0.01; ***p-value<0.001; ****p-value<0.0001).

## Slide 5
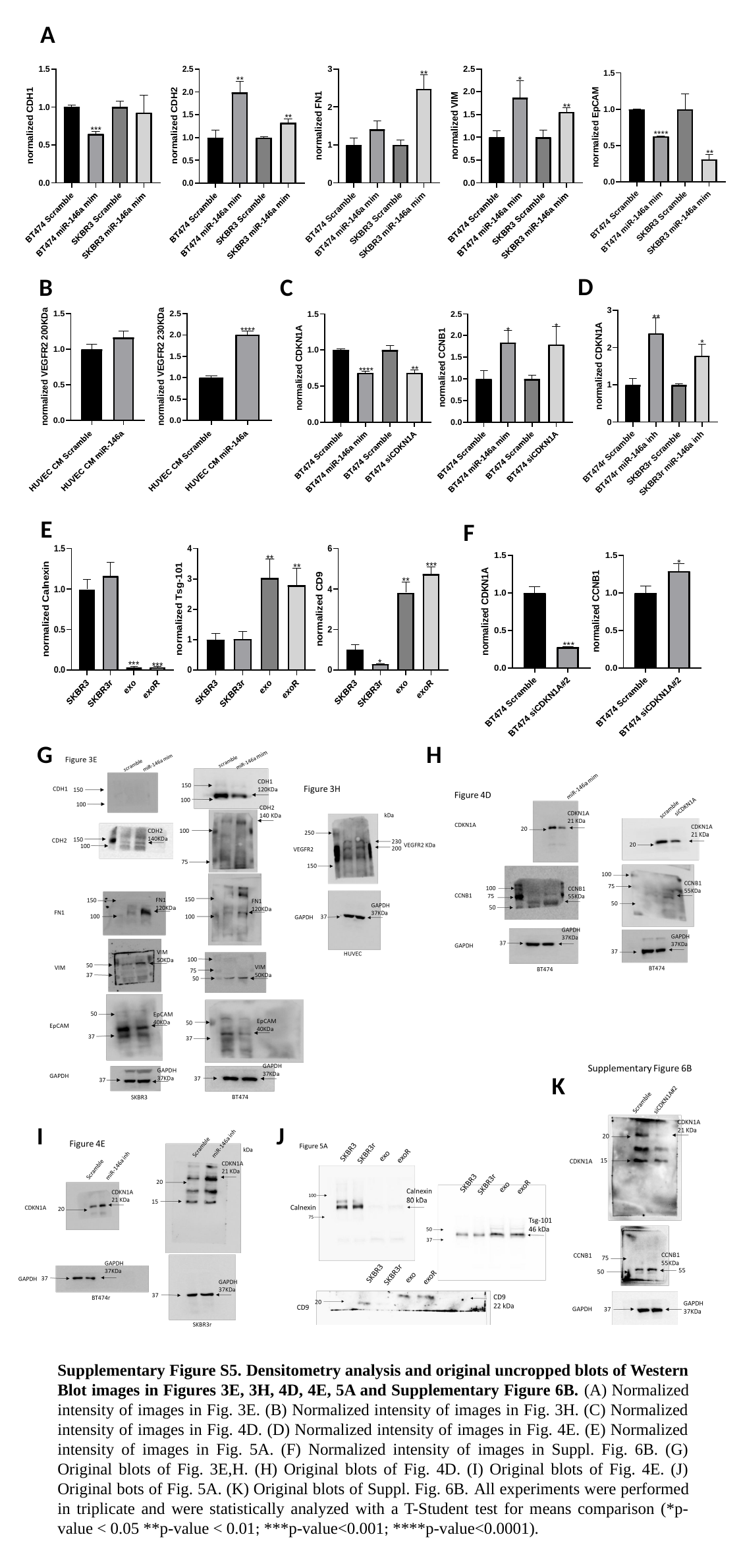

A
D
B
C
E
F
G
H
K
I
J
Supplementary Figure S5. Densitometry analysis and original uncropped blots of Western Blot images in Figures 3E, 3H, 4D, 4E, 5A and Supplementary Figure 6B. (A) Normalized intensity of images in Fig. 3E. (B) Normalized intensity of images in Fig. 3H. (C) Normalized intensity of images in Fig. 4D. (D) Normalized intensity of images in Fig. 4E. (E) Normalized intensity of images in Fig. 5A. (F) Normalized intensity of images in Suppl. Fig. 6B. (G) Original blots of Fig. 3E,H. (H) Original blots of Fig. 4D. (I) Original blots of Fig. 4E. (J) Original bots of Fig. 5A. (K) Original blots of Suppl. Fig. 6B. All experiments were performed in triplicate and were statistically analyzed with a T-Student test for means comparison (*p-value < 0.05 **p-value < 0.01; ***p-value<0.001; ****p-value<0.0001).

## Slide 6
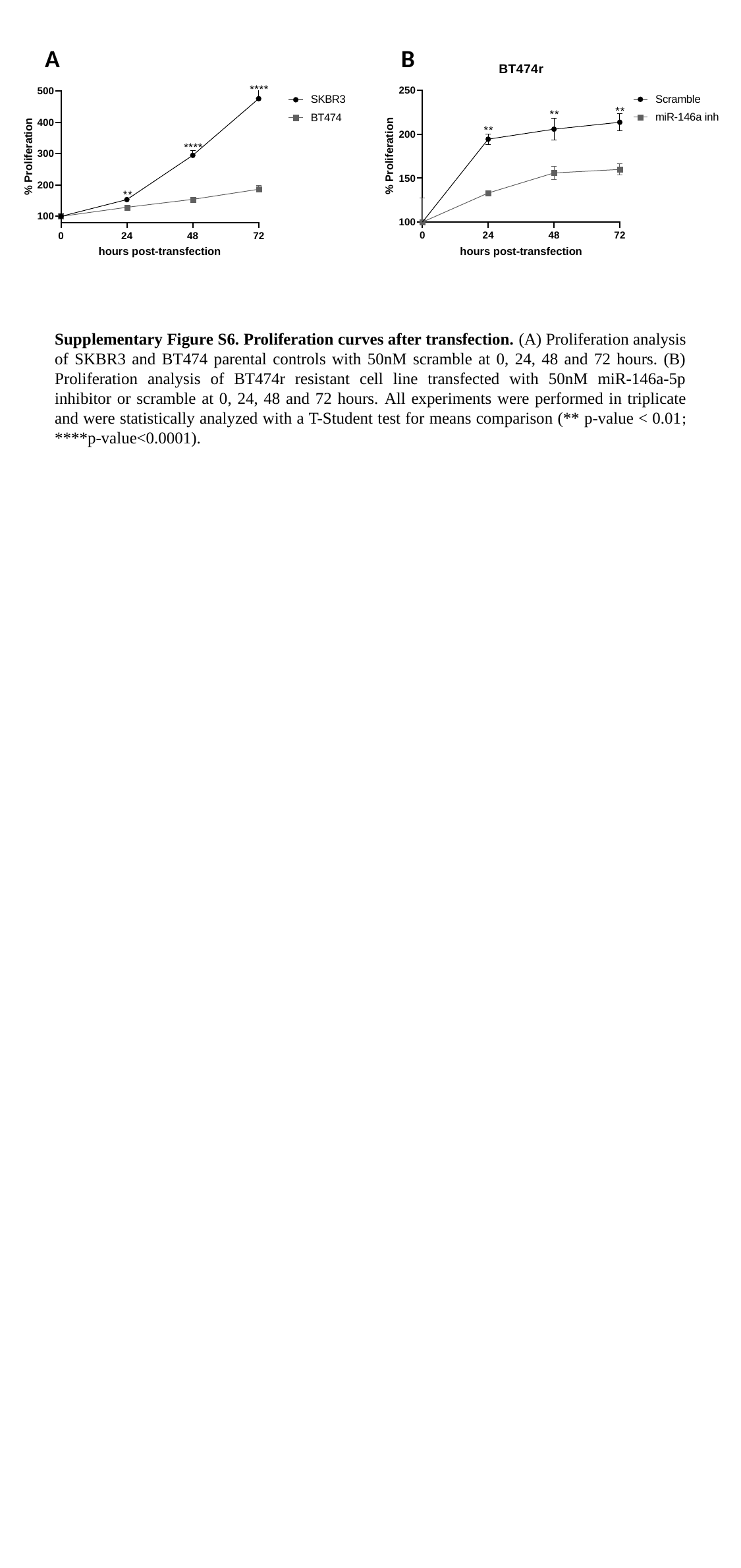

A
B
Supplementary Figure S6. Proliferation curves after transfection. (A) Proliferation analysis of SKBR3 and BT474 parental controls with 50nM scramble at 0, 24, 48 and 72 hours. (B) Proliferation analysis of BT474r resistant cell line transfected with 50nM miR-146a-5p inhibitor or scramble at 0, 24, 48 and 72 hours. All experiments were performed in triplicate and were statistically analyzed with a T-Student test for means comparison (** p-value < 0.01; ****p-value<0.0001).

## Slide 7
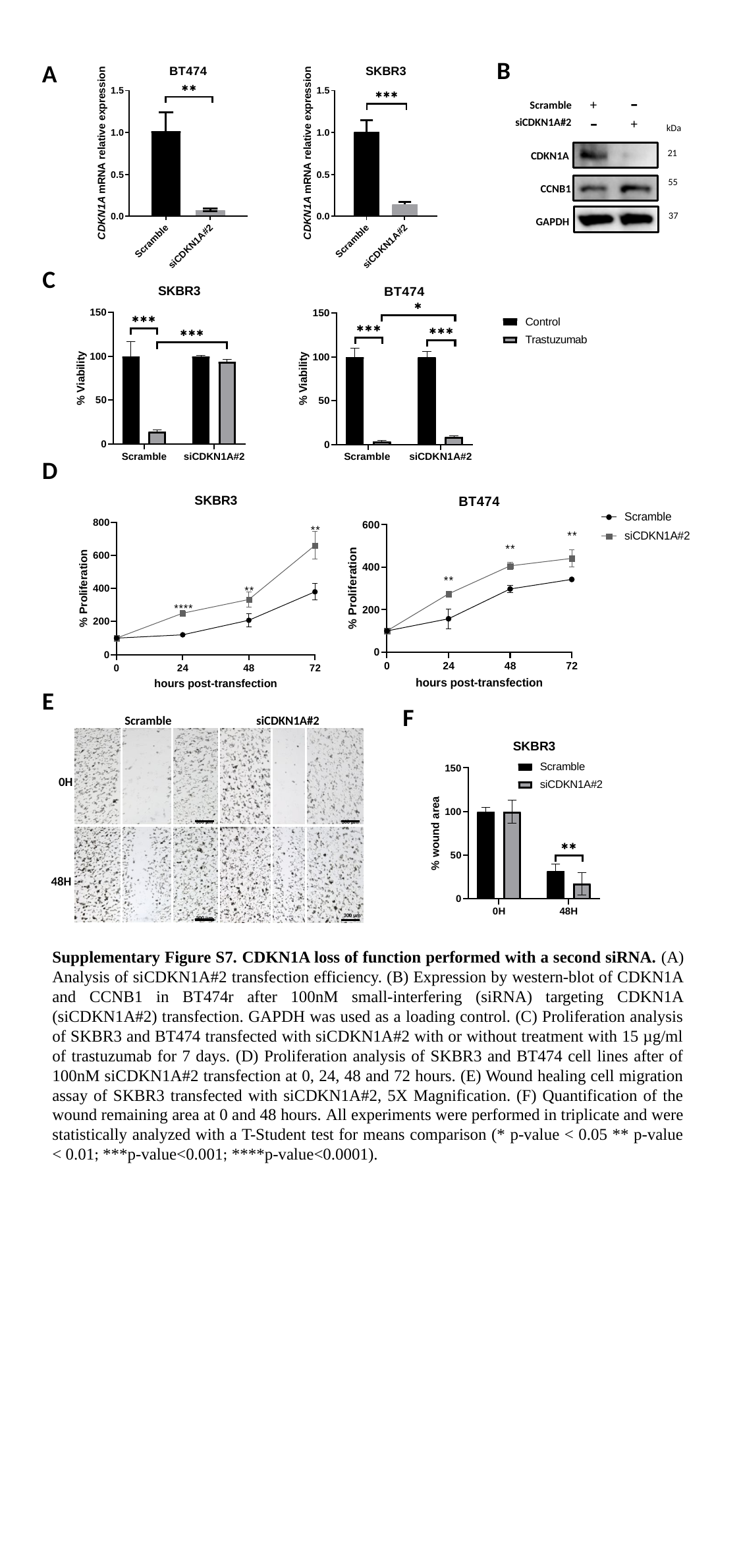

B
A
-
+
Scramble
-
siCDKN1A#2
+
kDa
21
CDKN1A
CCNB1
GAPDH
55
37
C
D
E
F
Scramble
siCDKN1A#2
0H
48H
300 μm
300 μm
300 μm
300 μm
Supplementary Figure S7. CDKN1A loss of function performed with a second siRNA. (A) Analysis of siCDKN1A#2 transfection efficiency. (B) Expression by western-blot of CDKN1A and CCNB1 in BT474r after 100nM small-interfering (siRNA) targeting CDKN1A (siCDKN1A#2) transfection. GAPDH was used as a loading control. (C) Proliferation analysis of SKBR3 and BT474 transfected with siCDKN1A#2 with or without treatment with 15 µg/ml of trastuzumab for 7 days. (D) Proliferation analysis of SKBR3 and BT474 cell lines after of 100nM siCDKN1A#2 transfection at 0, 24, 48 and 72 hours. (E) Wound healing cell migration assay of SKBR3 transfected with siCDKN1A#2, 5X Magnification. (F) Quantification of the wound remaining area at 0 and 48 hours. All experiments were performed in triplicate and were statistically analyzed with a T-Student test for means comparison (* p-value < 0.05 ** p-value < 0.01; ***p-value<0.001; ****p-value<0.0001).

## Slide 8
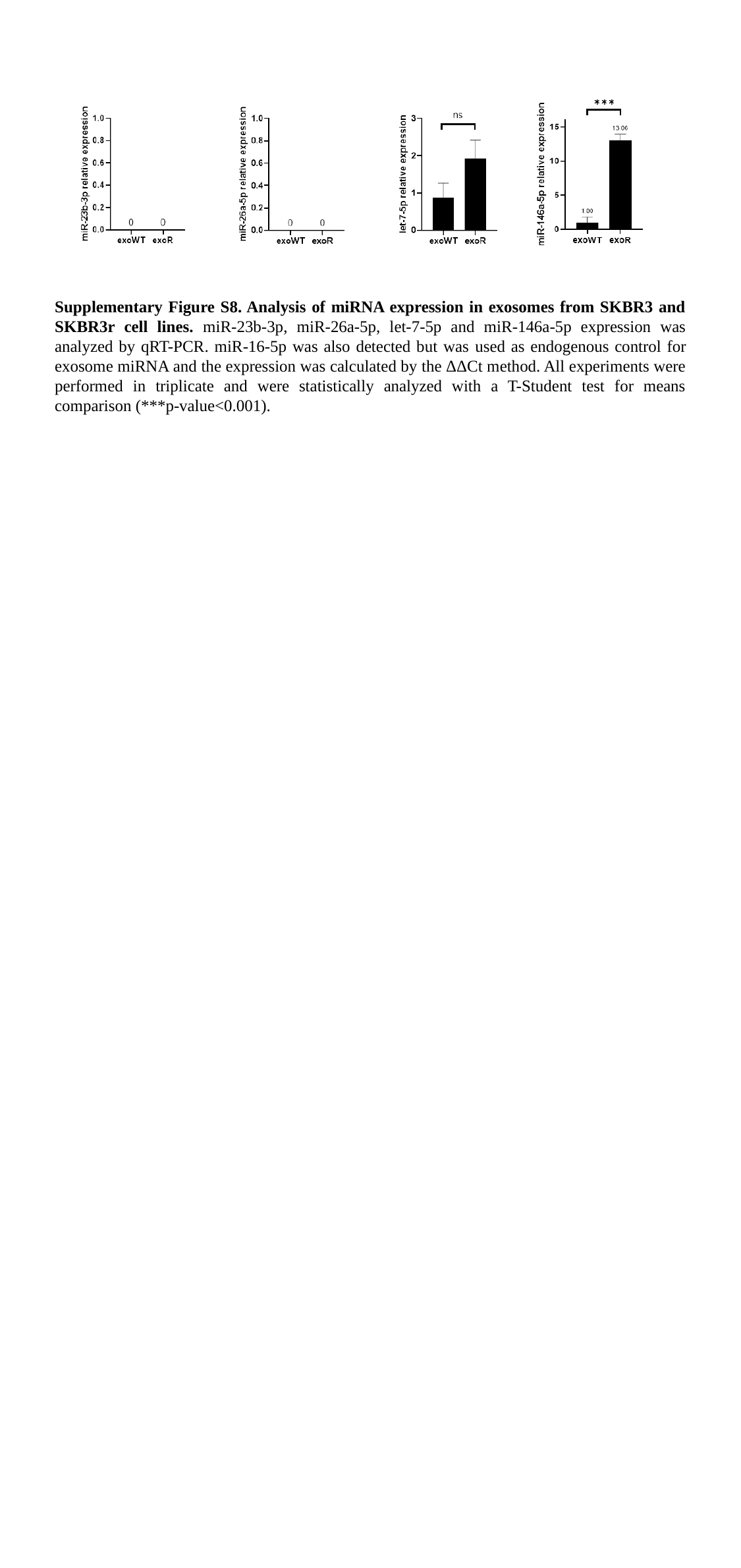

Supplementary Figure S8. Analysis of miRNA expression in exosomes from SKBR3 and SKBR3r cell lines. miR-23b-3p, miR-26a-5p, let-7-5p and miR-146a-5p expression was analyzed by qRT-PCR. miR-16-5p was also detected but was used as endogenous control for exosome miRNA and the expression was calculated by the ΔΔCt method. All experiments were performed in triplicate and were statistically analyzed with a T-Student test for means comparison (***p-value<0.001).

## Slide 9
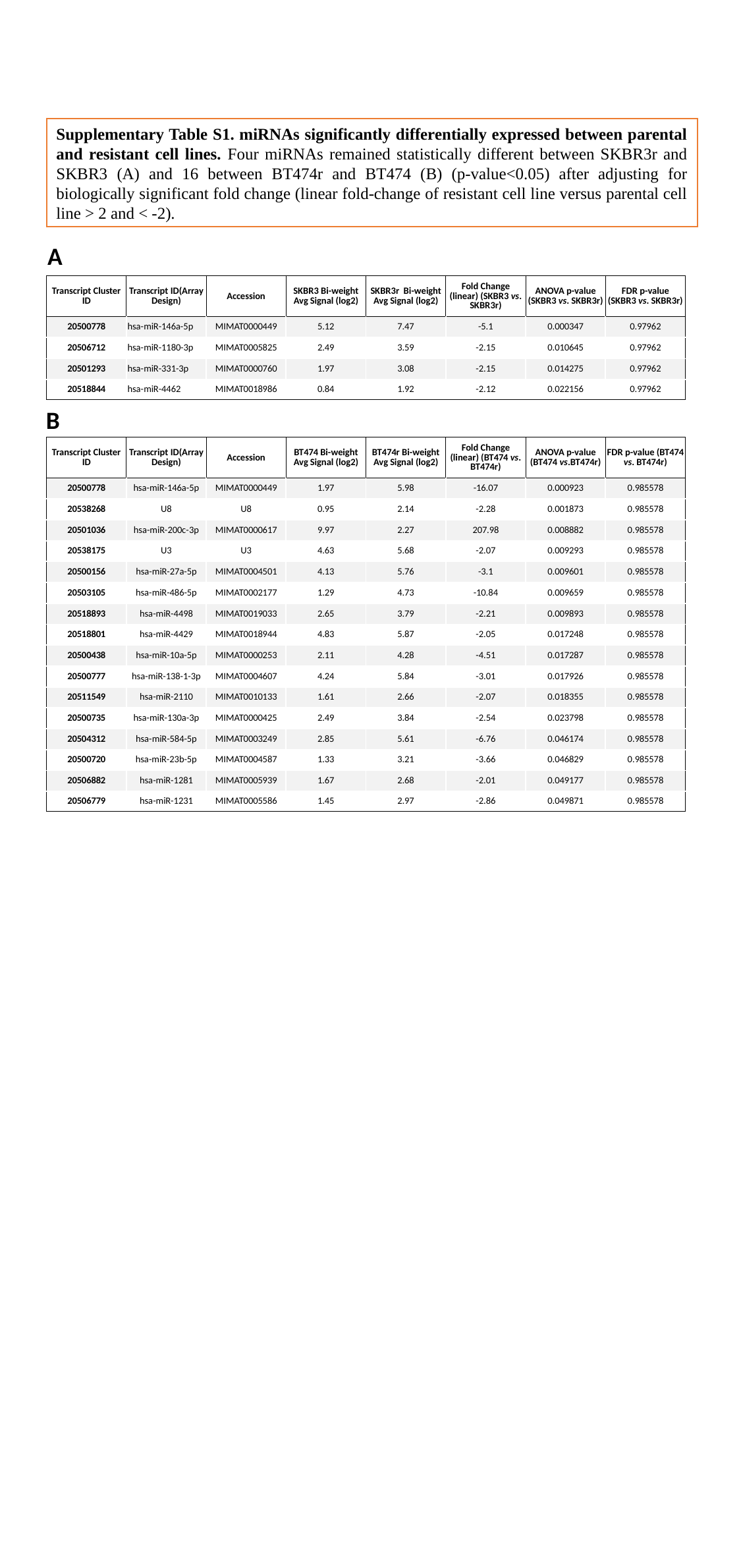

Supplementary Table S1. miRNAs significantly differentially expressed between parental and resistant cell lines. Four miRNAs remained statistically different between SKBR3r and SKBR3 (A) and 16 between BT474r and BT474 (B) (p-value<0.05) after adjusting for biologically significant fold change (linear fold-change of resistant cell line versus parental cell line > 2 and < -2).
A
| Transcript Cluster ID | Transcript ID(Array Design) | Accession | SKBR3 Bi-weight Avg Signal (log2) | SKBR3r Bi-weight Avg Signal (log2) | Fold Change (linear) (SKBR3 vs. SKBR3r) | ANOVA p-value (SKBR3 vs. SKBR3r) | FDR p-value (SKBR3 vs. SKBR3r) |
| --- | --- | --- | --- | --- | --- | --- | --- |
| 20500778 | hsa-miR-146a-5p | MIMAT0000449 | 5.12 | 7.47 | -5.1 | 0.000347 | 0.97962 |
| 20506712 | hsa-miR-1180-3p | MIMAT0005825 | 2.49 | 3.59 | -2.15 | 0.010645 | 0.97962 |
| 20501293 | hsa-miR-331-3p | MIMAT0000760 | 1.97 | 3.08 | -2.15 | 0.014275 | 0.97962 |
| 20518844 | hsa-miR-4462 | MIMAT0018986 | 0.84 | 1.92 | -2.12 | 0.022156 | 0.97962 |
B
| Transcript Cluster ID | Transcript ID(Array Design) | Accession | BT474 Bi-weight Avg Signal (log2) | BT474r Bi-weight Avg Signal (log2) | Fold Change (linear) (BT474 vs. BT474r) | ANOVA p-value (BT474 vs.BT474r) | FDR p-value (BT474 vs. BT474r) |
| --- | --- | --- | --- | --- | --- | --- | --- |
| 20500778 | hsa-miR-146a-5p | MIMAT0000449 | 1.97 | 5.98 | -16.07 | 0.000923 | 0.985578 |
| 20538268 | U8 | U8 | 0.95 | 2.14 | -2.28 | 0.001873 | 0.985578 |
| 20501036 | hsa-miR-200c-3p | MIMAT0000617 | 9.97 | 2.27 | 207.98 | 0.008882 | 0.985578 |
| 20538175 | U3 | U3 | 4.63 | 5.68 | -2.07 | 0.009293 | 0.985578 |
| 20500156 | hsa-miR-27a-5p | MIMAT0004501 | 4.13 | 5.76 | -3.1 | 0.009601 | 0.985578 |
| 20503105 | hsa-miR-486-5p | MIMAT0002177 | 1.29 | 4.73 | -10.84 | 0.009659 | 0.985578 |
| 20518893 | hsa-miR-4498 | MIMAT0019033 | 2.65 | 3.79 | -2.21 | 0.009893 | 0.985578 |
| 20518801 | hsa-miR-4429 | MIMAT0018944 | 4.83 | 5.87 | -2.05 | 0.017248 | 0.985578 |
| 20500438 | hsa-miR-10a-5p | MIMAT0000253 | 2.11 | 4.28 | -4.51 | 0.017287 | 0.985578 |
| 20500777 | hsa-miR-138-1-3p | MIMAT0004607 | 4.24 | 5.84 | -3.01 | 0.017926 | 0.985578 |
| 20511549 | hsa-miR-2110 | MIMAT0010133 | 1.61 | 2.66 | -2.07 | 0.018355 | 0.985578 |
| 20500735 | hsa-miR-130a-3p | MIMAT0000425 | 2.49 | 3.84 | -2.54 | 0.023798 | 0.985578 |
| 20504312 | hsa-miR-584-5p | MIMAT0003249 | 2.85 | 5.61 | -6.76 | 0.046174 | 0.985578 |
| 20500720 | hsa-miR-23b-5p | MIMAT0004587 | 1.33 | 3.21 | -3.66 | 0.046829 | 0.985578 |
| 20506882 | hsa-miR-1281 | MIMAT0005939 | 1.67 | 2.68 | -2.01 | 0.049177 | 0.985578 |
| 20506779 | hsa-miR-1231 | MIMAT0005586 | 1.45 | 2.97 | -2.86 | 0.049871 | 0.985578 |

## Slide 10
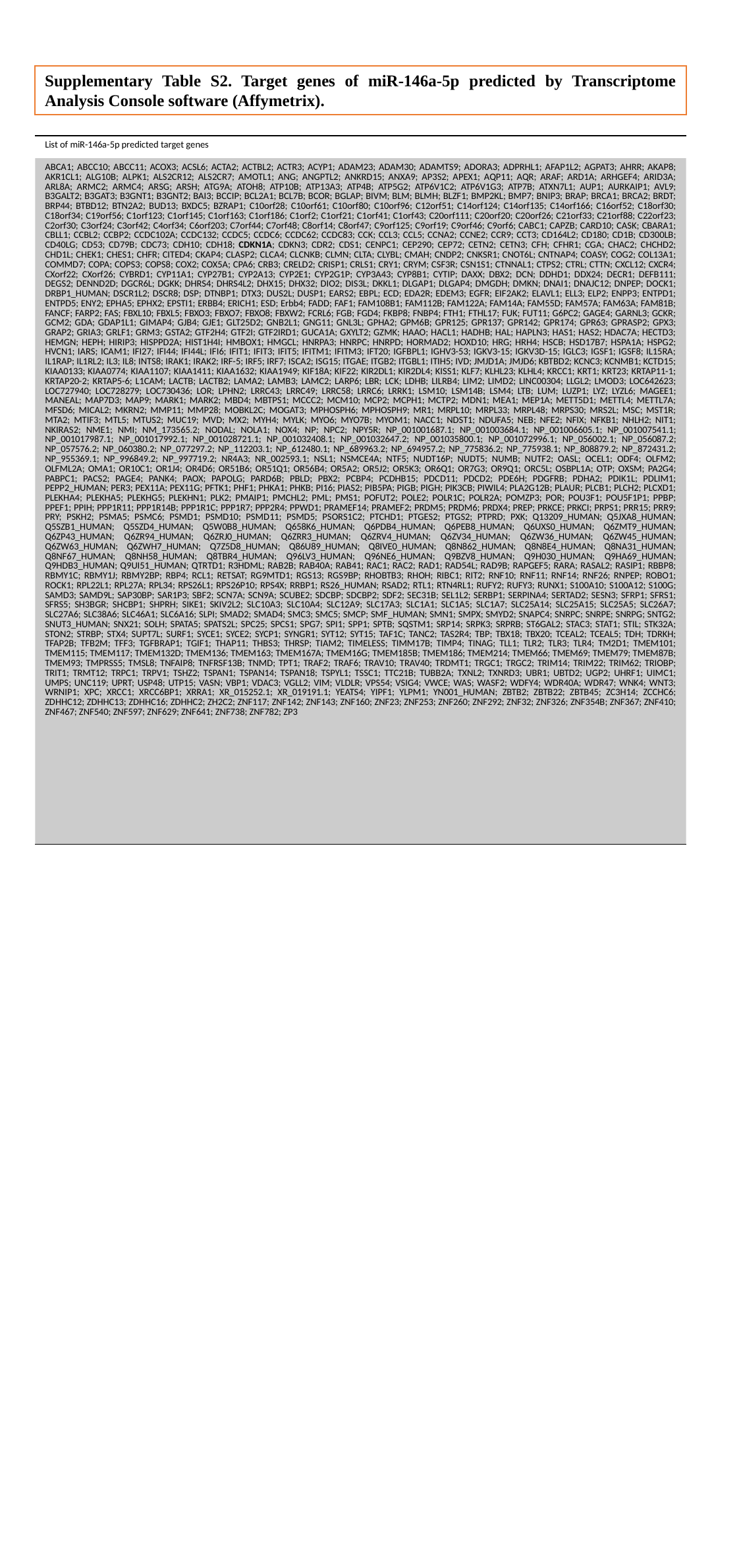

Supplementary Table S2. Target genes of miR-146a-5p predicted by Transcriptome Analysis Console software (Affymetrix).
| List of miR-146a-5p predicted target genes |
| --- |
| ABCA1; ABCC10; ABCC11; ACOX3; ACSL6; ACTA2; ACTBL2; ACTR3; ACYP1; ADAM23; ADAM30; ADAMTS9; ADORA3; ADPRHL1; AFAP1L2; AGPAT3; AHRR; AKAP8; AKR1CL1; ALG10B; ALPK1; ALS2CR12; ALS2CR7; AMOTL1; ANG; ANGPTL2; ANKRD15; ANXA9; AP3S2; APEX1; AQP11; AQR; ARAF; ARD1A; ARHGEF4; ARID3A; ARL8A; ARMC2; ARMC4; ARSG; ARSH; ATG9A; ATOH8; ATP10B; ATP13A3; ATP4B; ATP5G2; ATP6V1C2; ATP6V1G3; ATP7B; ATXN7L1; AUP1; AURKAIP1; AVL9; B3GALT2; B3GAT3; B3GNT1; B3GNT2; BAI3; BCCIP; BCL2A1; BCL7B; BCOR; BGLAP; BIVM; BLM; BLMH; BLZF1; BMP2KL; BMP7; BNIP3; BRAP; BRCA1; BRCA2; BRDT; BRP44; BTBD12; BTN2A2; BUD13; BXDC5; BZRAP1; C10orf28; C10orf61; C10orf80; C10orf96; C12orf51; C14orf124; C14orf135; C14orf166; C16orf52; C18orf30; C18orf34; C19orf56; C1orf123; C1orf145; C1orf163; C1orf186; C1orf2; C1orf21; C1orf41; C1orf43; C20orf111; C20orf20; C20orf26; C21orf33; C21orf88; C22orf23; C2orf30; C3orf24; C3orf42; C4orf34; C6orf203; C7orf44; C7orf48; C8orf14; C8orf47; C9orf125; C9orf19; C9orf46; C9orf6; CABC1; CAPZB; CARD10; CASK; CBARA1; CBLL1; CCBL2; CCBP2; CCDC102A; CCDC132; CCDC5; CCDC6; CCDC62; CCDC83; CCK; CCL3; CCL5; CCNA2; CCNE2; CCR9; CCT3; CD164L2; CD180; CD1B; CD300LB; CD40LG; CD53; CD79B; CDC73; CDH10; CDH18; CDKN1A; CDKN3; CDR2; CDS1; CENPC1; CEP290; CEP72; CETN2; CETN3; CFH; CFHR1; CGA; CHAC2; CHCHD2; CHD1L; CHEK1; CHES1; CHFR; CITED4; CKAP4; CLASP2; CLCA4; CLCNKB; CLMN; CLTA; CLYBL; CMAH; CNDP2; CNKSR1; CNOT6L; CNTNAP4; COASY; COG2; COL13A1; COMMD7; COPA; COPS3; COPS8; COX2; COX5A; CPA6; CRB3; CRELD2; CRISP1; CRLS1; CRY1; CRYM; CSF3R; CSN1S1; CTNNAL1; CTPS2; CTRL; CTTN; CXCL12; CXCR4; CXorf22; CXorf26; CYBRD1; CYP11A1; CYP27B1; CYP2A13; CYP2E1; CYP2G1P; CYP3A43; CYP8B1; CYTIP; DAXX; DBX2; DCN; DDHD1; DDX24; DECR1; DEFB111; DEGS2; DENND2D; DGCR6L; DGKK; DHRS4; DHRS4L2; DHX15; DHX32; DIO2; DIS3L; DKKL1; DLGAP1; DLGAP4; DMGDH; DMKN; DNAI1; DNAJC12; DNPEP; DOCK1; DRBP1\_HUMAN; DSCR1L2; DSCR8; DSP; DTNBP1; DTX3; DUS2L; DUSP1; EARS2; EBPL; ECD; EDA2R; EDEM3; EGFR; EIF2AK2; ELAVL1; ELL3; ELP2; ENPP3; ENTPD1; ENTPD5; ENY2; EPHA5; EPHX2; EPSTI1; ERBB4; ERICH1; ESD; Erbb4; FADD; FAF1; FAM108B1; FAM112B; FAM122A; FAM14A; FAM55D; FAM57A; FAM63A; FAM81B; FANCF; FARP2; FAS; FBXL10; FBXL5; FBXO3; FBXO7; FBXO8; FBXW2; FCRL6; FGB; FGD4; FKBP8; FNBP4; FTH1; FTHL17; FUK; FUT11; G6PC2; GAGE4; GARNL3; GCKR; GCM2; GDA; GDAP1L1; GIMAP4; GJB4; GJE1; GLT25D2; GNB2L1; GNG11; GNL3L; GPHA2; GPM6B; GPR125; GPR137; GPR142; GPR174; GPR63; GPRASP2; GPX3; GRAP2; GRIA3; GRLF1; GRM3; GSTA2; GTF2H4; GTF2I; GTF2IRD1; GUCA1A; GXYLT2; GZMK; HAAO; HACL1; HADHB; HAL; HAPLN3; HAS1; HAS2; HDAC7A; HECTD3; HEMGN; HEPH; HIRIP3; HISPPD2A; HIST1H4I; HMBOX1; HMGCL; HNRPA3; HNRPC; HNRPD; HORMAD2; HOXD10; HRG; HRH4; HSCB; HSD17B7; HSPA1A; HSPG2; HVCN1; IARS; ICAM1; IFI27; IFI44; IFI44L; IFI6; IFIT1; IFIT3; IFIT5; IFITM1; IFITM3; IFT20; IGFBPL1; IGHV3-53; IGKV3-15; IGKV3D-15; IGLC3; IGSF1; IGSF8; IL15RA; IL1RAP; IL1RL2; IL3; IL8; INTS8; IRAK1; IRAK2; IRF-5; IRF5; IRF7; ISCA2; ISG15; ITGAE; ITGB2; ITGBL1; ITIH5; IVD; JMJD1A; JMJD6; KBTBD2; KCNC3; KCNMB1; KCTD15; KIAA0133; KIAA0774; KIAA1107; KIAA1411; KIAA1632; KIAA1949; KIF18A; KIF22; KIR2DL1; KIR2DL4; KISS1; KLF7; KLHL23; KLHL4; KRCC1; KRT1; KRT23; KRTAP11-1; KRTAP20-2; KRTAP5-6; L1CAM; LACTB; LACTB2; LAMA2; LAMB3; LAMC2; LARP6; LBR; LCK; LDHB; LILRB4; LIM2; LIMD2; LINC00304; LLGL2; LMOD3; LOC642623; LOC727940; LOC728279; LOC730436; LOR; LPHN2; LRRC43; LRRC49; LRRC58; LRRC6; LRRK1; LSM10; LSM14B; LSM4; LTB; LUM; LUZP1; LYZ; LYZL6; MAGEE1; MANEAL; MAP7D3; MAP9; MARK1; MARK2; MBD4; MBTPS1; MCCC2; MCM10; MCP2; MCPH1; MCTP2; MDN1; MEA1; MEP1A; METT5D1; METTL4; METTL7A; MFSD6; MICAL2; MKRN2; MMP11; MMP28; MOBKL2C; MOGAT3; MPHOSPH6; MPHOSPH9; MR1; MRPL10; MRPL33; MRPL48; MRPS30; MRS2L; MSC; MST1R; MTA2; MTIF3; MTL5; MTUS2; MUC19; MVD; MX2; MYH4; MYLK; MYO6; MYO7B; MYOM1; NACC1; NDST1; NDUFA5; NEB; NFE2; NFIX; NFKB1; NHLH2; NIT1; NKIRAS2; NME1; NMI; NM\_173565.2; NODAL; NOLA1; NOX4; NP; NPC2; NPY5R; NP\_001001687.1; NP\_001003684.1; NP\_001006605.1; NP\_001007541.1; NP\_001017987.1; NP\_001017992.1; NP\_001028721.1; NP\_001032408.1; NP\_001032647.2; NP\_001035800.1; NP\_001072996.1; NP\_056002.1; NP\_056087.2; NP\_057576.2; NP\_060380.2; NP\_077297.2; NP\_112203.1; NP\_612480.1; NP\_689963.2; NP\_694957.2; NP\_775836.2; NP\_775938.1; NP\_808879.2; NP\_872431.2; NP\_955369.1; NP\_996849.2; NP\_997719.2; NR4A3; NR\_002593.1; NSL1; NSMCE4A; NTF5; NUDT16P; NUDT5; NUMB; NUTF2; OASL; OCEL1; ODF4; OLFM2; OLFML2A; OMA1; OR10C1; OR1J4; OR4D6; OR51B6; OR51Q1; OR56B4; OR5A2; OR5J2; OR5K3; OR6Q1; OR7G3; OR9Q1; ORC5L; OSBPL1A; OTP; OXSM; PA2G4; PABPC1; PACS2; PAGE4; PANK4; PAOX; PAPOLG; PARD6B; PBLD; PBX2; PCBP4; PCDHB15; PDCD11; PDCD2; PDE6H; PDGFRB; PDHA2; PDIK1L; PDLIM1; PEPP2\_HUMAN; PER3; PEX11A; PEX11G; PFTK1; PHF1; PHKA1; PHKB; PI16; PIAS2; PIB5PA; PIGB; PIGH; PIK3CB; PIWIL4; PLA2G12B; PLAUR; PLCB1; PLCH2; PLCXD1; PLEKHA4; PLEKHA5; PLEKHG5; PLEKHN1; PLK2; PMAIP1; PMCHL2; PML; PMS1; POFUT2; POLE2; POLR1C; POLR2A; POMZP3; POR; POU3F1; POU5F1P1; PPBP; PPEF1; PPIH; PPP1R11; PPP1R14B; PPP1R1C; PPP1R7; PPP2R4; PPWD1; PRAMEF14; PRAMEF2; PRDM5; PRDM6; PRDX4; PREP; PRKCE; PRKCI; PRPS1; PRR15; PRR9; PRY; PSKH2; PSMA5; PSMC6; PSMD1; PSMD10; PSMD11; PSMD5; PSORS1C2; PTCHD1; PTGES2; PTGS2; PTPRD; PXK; Q13209\_HUMAN; Q5JXA8\_HUMAN; Q5SZB1\_HUMAN; Q5SZD4\_HUMAN; Q5W0B8\_HUMAN; Q658K6\_HUMAN; Q6PDB4\_HUMAN; Q6PEB8\_HUMAN; Q6UXS0\_HUMAN; Q6ZMT9\_HUMAN; Q6ZP43\_HUMAN; Q6ZR94\_HUMAN; Q6ZRJ0\_HUMAN; Q6ZRR3\_HUMAN; Q6ZRV4\_HUMAN; Q6ZV34\_HUMAN; Q6ZW36\_HUMAN; Q6ZW45\_HUMAN; Q6ZW63\_HUMAN; Q6ZWH7\_HUMAN; Q7Z5D8\_HUMAN; Q86U89\_HUMAN; Q8IVE0\_HUMAN; Q8N862\_HUMAN; Q8N8E4\_HUMAN; Q8NA31\_HUMAN; Q8NF67\_HUMAN; Q8NH58\_HUMAN; Q8TBR4\_HUMAN; Q96LV3\_HUMAN; Q96NE6\_HUMAN; Q9BZV8\_HUMAN; Q9H030\_HUMAN; Q9HA69\_HUMAN; Q9HDB3\_HUMAN; Q9UI51\_HUMAN; QTRTD1; R3HDML; RAB2B; RAB40A; RAB41; RAC1; RAC2; RAD1; RAD54L; RAD9B; RAPGEF5; RARA; RASAL2; RASIP1; RBBP8; RBMY1C; RBMY1J; RBMY2BP; RBP4; RCL1; RETSAT; RG9MTD1; RGS13; RGS9BP; RHOBTB3; RHOH; RIBC1; RIT2; RNF10; RNF11; RNF14; RNF26; RNPEP; ROBO1; ROCK1; RPL22L1; RPL27A; RPL34; RPS26L1; RPS26P10; RPS4X; RRBP1; RS26\_HUMAN; RSAD2; RTL1; RTN4RL1; RUFY2; RUFY3; RUNX1; S100A10; S100A12; S100G; SAMD3; SAMD9L; SAP30BP; SAR1P3; SBF2; SCN7A; SCN9A; SCUBE2; SDCBP; SDCBP2; SDF2; SEC31B; SEL1L2; SERBP1; SERPINA4; SERTAD2; SESN3; SFRP1; SFRS1; SFRS5; SH3BGR; SHCBP1; SHPRH; SIKE1; SKIV2L2; SLC10A3; SLC10A4; SLC12A9; SLC17A3; SLC1A1; SLC1A5; SLC1A7; SLC25A14; SLC25A15; SLC25A5; SLC26A7; SLC27A6; SLC38A6; SLC46A1; SLC6A16; SLPI; SMAD2; SMAD4; SMC3; SMC5; SMCP; SMF\_HUMAN; SMN1; SMPX; SMYD2; SNAPC4; SNRPC; SNRPE; SNRPG; SNTG2; SNUT3\_HUMAN; SNX21; SOLH; SPATA5; SPATS2L; SPC25; SPCS1; SPG7; SPI1; SPP1; SPTB; SQSTM1; SRP14; SRPK3; SRPRB; ST6GAL2; STAC3; STAT1; STIL; STK32A; STON2; STRBP; STX4; SUPT7L; SURF1; SYCE1; SYCE2; SYCP1; SYNGR1; SYT12; SYT15; TAF1C; TANC2; TAS2R4; TBP; TBX18; TBX20; TCEAL2; TCEAL5; TDH; TDRKH; TFAP2B; TFB2M; TFF3; TGFBRAP1; TGIF1; THAP11; THBS3; THRSP; TIAM2; TIMELESS; TIMM17B; TIMP4; TINAG; TLL1; TLR2; TLR3; TLR4; TM2D1; TMEM101; TMEM115; TMEM117; TMEM132D; TMEM136; TMEM163; TMEM167A; TMEM16G; TMEM185B; TMEM186; TMEM214; TMEM66; TMEM69; TMEM79; TMEM87B; TMEM93; TMPRSS5; TMSL8; TNFAIP8; TNFRSF13B; TNMD; TPT1; TRAF2; TRAF6; TRAV10; TRAV40; TRDMT1; TRGC1; TRGC2; TRIM14; TRIM22; TRIM62; TRIOBP; TRIT1; TRMT12; TRPC1; TRPV1; TSHZ2; TSPAN1; TSPAN14; TSPAN18; TSPYL1; TSSC1; TTC21B; TUBB2A; TXNL2; TXNRD3; UBR1; UBTD2; UGP2; UHRF1; UIMC1; UMPS; UNC119; UPRT; USP48; UTP15; VASN; VBP1; VDAC3; VGLL2; VIM; VLDLR; VPS54; VSIG4; VWCE; WAS; WASF2; WDFY4; WDR40A; WDR47; WNK4; WNT3; WRNIP1; XPC; XRCC1; XRCC6BP1; XRRA1; XR\_015252.1; XR\_019191.1; YEATS4; YIPF1; YLPM1; YN001\_HUMAN; ZBTB2; ZBTB22; ZBTB45; ZC3H14; ZCCHC6; ZDHHC12; ZDHHC13; ZDHHC16; ZDHHC2; ZH2C2; ZNF117; ZNF142; ZNF143; ZNF160; ZNF23; ZNF253; ZNF260; ZNF292; ZNF32; ZNF326; ZNF354B; ZNF367; ZNF410; ZNF467; ZNF540; ZNF597; ZNF629; ZNF641; ZNF738; ZNF782; ZP3 |
